# Spatial Configuration of Abdominal Aortic Aneurysm Analysis as a Useful Tool for the Estimation of Stent-Graft Migration

**DOI:** 10.3390/diagnostics10100737

**Published:** 2020-09-23

**Authors:** Andrzej Polanczyk, Aleksandra Piechota-Polanczyk, Ludomir Stefańczyk, Michał Strzelecki

**Affiliations:** 1 The Main School of Fire Service, 01-629 Warsaw, Poland; 2 Department of Medical Biotechnology, Jagiellonian University, 30-387 Krakow, Poland; aleksandra.piechota-polanczyk@uj.edu.pl; 3 Department of Radiology and Diagnostic Imaging, Medical University of Lodz, 90-153 Lodz, Poland; ludomir.stefanczyk@umed.lodz.pl; 4 Institute of Electronics, Lodz University of Technology, 93-005 Lodz, Poland; michal.strzelecki@p.lodz.pl

**Keywords:** shape factor, shape analysis, aneurysm, endovascular prothesis

## Abstract

The aim of this study was to prepare a self-made mathematical algorithm for the estimation of risk of stent-graft migration with the use of data on abdominal aortic aneurysm (AAA) size and geometry of blood flow through aneurysm sac before or after stent-graft implantation. AngioCT data from 20 patients aged 50–60 years, before and after stent-graft placement in the AAA was analyzed. In order to estimate the risk of stent-graft migration for each patient we prepared an opposite spatial configuration of virtually reconstructed stent-graft with long body or short body. Thus, three groups of 3D geometries were analyzed: 20 geometries representing 3D models of aneurysm, 20 geometries representing 3D models of long body stent-grafts, and 20 geometries representing 3D models of short body stent-graft. The proposed self-made algorithm demonstrated its efficiency and usefulness in estimating wall shear stress (WSS) values. Comparison of the long or short type of stent-graft with AAA geometries allowed to analyze the implants’ spatial configuration. Our study indicated that short stent-graft, after placement in the AAA sac, generated lower drug forces compare to the long stent-graft. Each time shape factor was higher for short stent-graft compare to long stent-graft.

## 1. Introduction

The XX and XXI centuries are characterized by prolongation of life span and increasing number of people diagnosed with an abdominal aortic aneurysm (AAA), that occurs in 5% of the society elder that 65 years of age [1]. AAA is a high-risk vascular disease which treatment depends on its diameter. When the diameter is lower than 40 mm pharmacological treatment is applied [2,3], while AAA with diameter equal or above 55 mm and growth rate over 5 mm every 6 months require surgical repair either open or endovascular [4].

Endovascular aortic aneurysm repair (EVAR) is “less” invasive and characterized by lower postoperative complications and mortality rate [5]. However, potential complications of EVAR, such as endoleaks, migration or appearance of angular bands in the stent-graft (SG) body or legs, have raised concerns about its durability. More severe complications include stent-graft lumen stenosis and occlusion. Clot formation can be a consequence of improper SG geometry or the appearance of angular bends [6]. Computational tomography (CT) and magnetic resonance angiography (MRA) are useful tools for diagnostic purposes because they can detect thrombus both inside the endograft and native iliac vessels. However, both medical imaging modalities cannot depict the local hemodynamics. Although useful for diagnostic procedures Angio-CT can only detect clot when it appears inside SG [7,8]. The use of numerical methods, e.g., computational fluid dynamic (CFD) technique in solving problems related to blood hemodynamic in cardiac system is widely described in the literature [9,10,11,12]. CFD technique is most often performed with the use of medical imaging like Angio-CT, MRI or X-Ray [13,14,15]. The real geometry of a blood vessel is usually obtained using medical data acquired from Angio-CT or MRI [16]. Recently, many studies connected with CFD techniques are orientated on assessing blood hemodynamic in vessels after stent-graft’s placement [17], and on a spatial configuration of endovascular implants [18,19]. Depending on the type of analyzed problem, blood is treated as either a Newtonian [20,21] or non-Newtonian fluid [22,23].

There are situations when systemic conditions make the comparison of homogeneous groups of operated patients a complicated task. Routine use of endovascular prosthesis has been advocated to reduce restenosis, stroke, and death, but its protective effect, particularly from late restenosis, is less evident and recent studies call into question this thesis. Hence, it seems promising to use CFD technique with further verification in clinical observations. However, even then the comparison of gathered results and their implementation to other patients is difficult [24]. Thus, it is essential to analyze blood hemodynamic inside AAA or stent-graft [25]. While, evaluation of wall shear stress (WSS) in AAA before and after stent-graft placement with conventional methods maybe impossible, CFD technique appears as a valid alternative option [26]. Therefore, we aimed to prepare a non-invasive quantitative tool for radiologists and surgeons for the characterization of blood hemodynamic in the area of AAA before and after stent-graft placement and the value of drag force acting on the endovascular prosthesis after placement inside AAA. This is a unique study where we use medical data not to plan the surgery but to predict its outcome. The accuracy of the proposed approach will be demonstrated and the reliability of obtained results will be verified with medical data.

The paper is organized as follows: In Section II, medical data, a mathematical model with boundary conditions and its verification is described. Section III presents the results directed in the computer simulation, shape factor application, and relation between aneurysm and stent-graft for the risk of migration estimation. In Section IV a discussion is proposed, while section V concludes the paper.

## 2. Materials and Methods

In this study we used AngioCT data (GE Light-Speed 64 VCT; GE Healthcare, Fairfield, CT, USA) from 20 patients aged 50–60 years, before and after stent-graft placement in the AAA. Patients were supplied with Zenith stent-graft made by COOK (Cook Medical, Bloomington, IN, USA). Contrast was included in the radiological diagnosis. As the total concentration of contrast (Visipaque) during one AngioCT analysis was constant (1.5 mL per 1 kg of body weight) in the aorta, we focused only on the distribution of brightness in whole domain for the aorta’s reconstruction [27]. Medical data was retrospectively collected after obtaining written informed consent to participate in the study and were anonymized before analysis. The study was approved by the Local Ethic Committee on Medical University of Lodz (RNN/126/07/KE, 20 March 2007).

Three-dimensional models of AAA before and after stent-graft placement were reconstructed similarly to previously described in our papers [14,28]. For the recognition of abdominal aortic aneurysm and implanted stent-graft Digital Imaging and Communications in Medicine (DICOM) data (512 × 512 × 270 voxels, in-plane resolution of 0.78 × 0.78 mm, slice thickness 0.8 mm) from the aforementioned patients were used. Each time DICOM data was applied to extract a model of stent-graft or AAA to generate the surface object, stored in the stereolithography (STL) format. To achieve the highest contrast between analyzed objects, AAA or stent-graft, and surrounding tissue AngioCT data was manually adjusted for brightness. Moreover, to extract AAA or stent-graft from the background the region growing technique was applied. ImageJ software and its tool for morphological holes filling was used for gaps elimination. The implemented segmentation region growing technique provided accurate results, since the AAA gray levels differed significantly from the image background. When compared to manual segmentation performed by the radiologist the estimated AAA and implanted stent-grafts did not differ more than by 5%. Each time to reconstruct 3D model of AAA as well as implanted stent-graft after segmentation process, a rendering process was performed. Moreover, a quantitative analysis of AngioCT data following [29] was used. At first brightness intensity to noise (BI) as a quotient of analyzed object brightness intensity and noise value was calculated. Secondly, contrast to noise ratio (CNR) as a quotient of subtraction of analyzed object brightness intensity and background brightness to noise value was calculated [30]. The highest brightness intensity was calculated in Pixels by placing Region of Interest (ROI) in the center of the area represented by analyzed AAA or stent-graft (reaching 80 mm^2^). This operation was performed for all slices for particular patient. The mean of these values was used for further calculations. While image noise was calculated as a ratio of ROI mean and standard deviation measured as well in pixels and calculated for 100 mm^2^ drawn in two different regions outside the patient body (left, and right sides). Average brightness intensity to noise was equal to 24.90, while average contrast to noise ratios was equal to 4.38.

**Figure 1:**
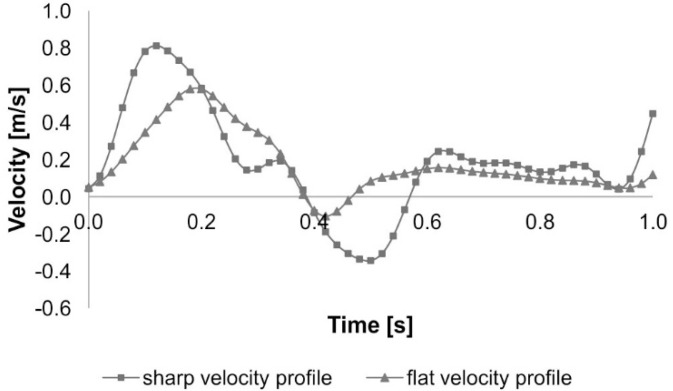
Sharp and flat reference of blood velocity profiles.

In order to estimate the risk of stent-graft migration for each patient we prepared an opposite spatial configuration of virtually reconstructed stent-graft with long body or short body [31,32]. Thus, three groups of 3D geometries were analyzed: 20 geometries representing 3D models of aneurysms, 20 geometries representing 3D models of long body stent-grafts, and 20 geometries representing 3D models of short body stent-grafts [13]. Next, with the use of pre-processor ANSYS ICEM CFD (ANSYS, Canonsburg, PA USA) numerical grids composed of 300,000 to 500,000 tetrahedral elements (according to mesh independent tests) were created [33]. Blood flow was calculated with the use of Ansys Fluent 17.1 software (ANSYS, Canonsburg, PA USA), using Euler method for solving Navier–Stokes equations. We assumed that the blood flow was incompressible and laminar and used Dirichlet conditions for the mathematical model of this flow. The following boundary conditions were applied: Velocity inlet [$\vec{v}$ (x, y, z)], *p* = const at the outlets from the geometry and rigid wall (fluid-solid interface, the boundary condition v = 0 was used) [34]. Following Johnston et al. blood was treated as non-Newtonian liquid [35]. Blood rheology was performed with the use of Quemada’s model as previously described [11]. Moreover, Quemada’s model includes initial parameters such as hematocrit (Hct), which was around 40% in the described patients. Therefore, blood hematocrit included in CFD model for the analyzed patients was 40%. Furthermore, blood density had a set value which enabled us to treat it as incompressible liquid (ρ = const). The blood velocity profiles for each of analyzed patients were obtained from the USG-Doppler examination (GE Vivid 7, GE Healthcare, Fairfield, CT, USA) [36,37]. Moreover, to standardize the 3D models of AAA and stent-grafts reference velocity profiles, flat and sharp, were applied (Figure 1) [38]. Therefore, the risk of stent-graft migration was estimated each time for three hemodynamic conditions: Real blood flow, and two extreme hemodynamic cases.

**Figure 2:**
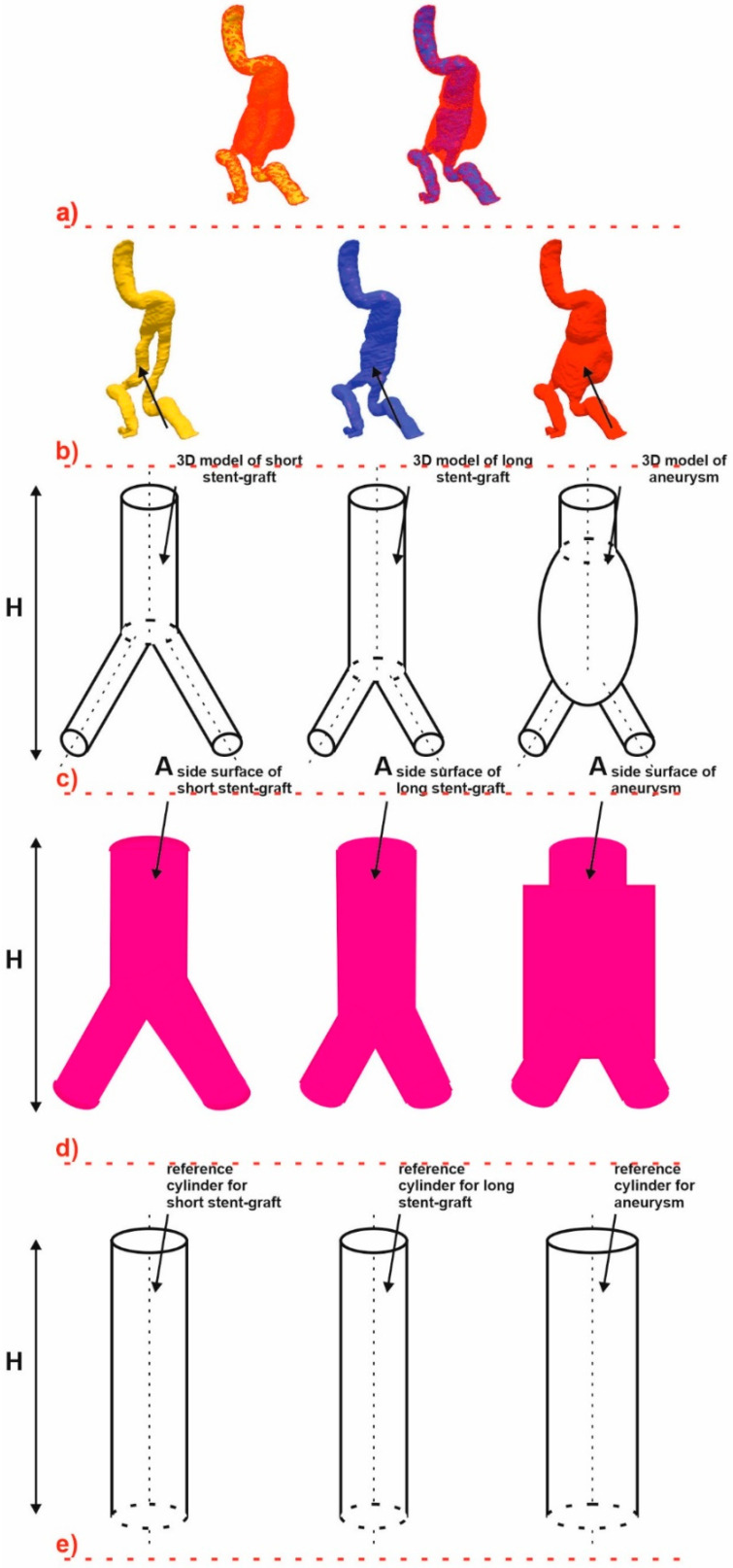
Diagram of shape factor calculation. (**a**)—reference 3D geometries of abdominal aortic aneurysm (AAA) with long and short stent-graft; (**b**)—reference 3D geometries of long and short stent-graft and AAA; (**c**)—a schema of long and short stent-graft and AAA; (**d**)—a schema of side surface of long and short stent-graft and AAA; (**e**)—a schema or reference cylinders of long and short stent-graft and AAA.

Spatial configuration of 3D geometries was calculated with the use of shape factor which we previously described [31,32]. However, this approach did not include the relation between spatial configuration of AAA and endovascular prosthesis. Therefore, we proposed a modification of the previous algorithm which included preparation of two reference cylinders based on aneurysm and endovascular implant geometries (Figure 2).

Each time 3D geometries including AAA with different spatial configuration of stent-graft inside was reconstructed (Figure 2a). One algorithm represented applied stent-graft and the other supplied area of aneurysm (Figure 2b). Therefore, each time object’s height was assumed to be the same and equal to the distance of supplied aneurysm (Equation (1)) (Figure 2c).

h_aneurysm_ = h_stent-graft_ (1)

where: h_aneurysm_—height equal to distance of supplied aneurysm, [m]; h_stent-graft_—height of analyzed stent-graft, [m].

In the first step a reference cylinder diameter was calculated from aneurysm side surface and stent-graft side surface (with constant high assumption, Equation (1)) (Figure 2d). Next, for each analyzed object such as AAA and stent-graft inside, reference cylinders were calculated (diameter, height and side surface) (Figure 2e). Finally, according to the above assumptions, the final shape factor describing spatial relation between aneurysm and stent-graft was described with Equation (2).

φ_A-S_ = A_aneurysm_/A_stent-graft_ (2)

where: A_aneurysm_—reference side surface of aneurysm, [m^3^]; A_stent-graft_—reference side surface of stent-graft, [m^3^].

As mechanical properties depend on the size of object’s surface, we proposed a modification of a shape factor algorithm. In the first step we calculated wall shear stress (WSS) (Equation (3)).

WSS = →∫ F dA(3)

where: WSS—shear stress on stent-graft wall, [Pa]; F—force acting on a side surface of a stent-graft, [N]; A—side surface of an aneurysm or stent-graft, [m^2^].

The total WSS value in whole cardiac cycle, representing total drag force acting on stent-graft’s wall, was calculated as time integral of the instantaneous WSS values (Equation (4)).

(4) $$WSS_{tot}\;=\;\overset{n}{\underset{k\;=\;1}{\sum }}\frac{WSS\left({\Delta t}_{k}\right)}{n}\;=\;\frac{1}{n}\overset{n}{\underset{k\;=\;1}{\sum }}WSS\left({\Delta t}_{k}\right)$$

where: *WSS_tot_*—total shear stress on stent-graft wall, [Pa]; Δt*_k_* = Δt for all *k*, [s]; *n*—number of time steps, [-].

Finally, it was possible to calculate a WSS factor that combined WSS recorded for aneurysm and stent-graft placed inside aneurysm (Equation (5)).

φ_WSS_ = WSS_aneurysm_/WSS_stent-graft_ (5)

where: φ_WSS_—wall shear factor, [-]; WSS_stent-graft_—wall shear stress acting on side surface of aneurysm supplied with stent-graft, [Pa]; WSS_aneurysm_—wall shear stress acting on stent-graft, n-type stent-graft (SL—long body, SS—short body), [Pa].

Comparison of drag forces of two spatial configurations of the same stent-graft, long body, and short body for the same patient allowed to indicate the correct geometry, presenting the lowest risk of migration. Each time we had a patient with one spatial configuration of stent-graft (long or short body) and the missing one was created artificially. Next, CFD results were verified with the medical data, where patients with and without diagnosed stent-graft movement were applied.

Statistical analysis was performed using GraphPad Prism Version 5.01 (GraphPad Software; San Diego, CA, USA). Values were presented as mean ± SEM. Correlations were evaluated with the Spearman rank correlation test or Pearson test. Data were considered as significantly different when *p* < 0.05 unless otherwise noted.

## 3. Results

**Figure 3:**
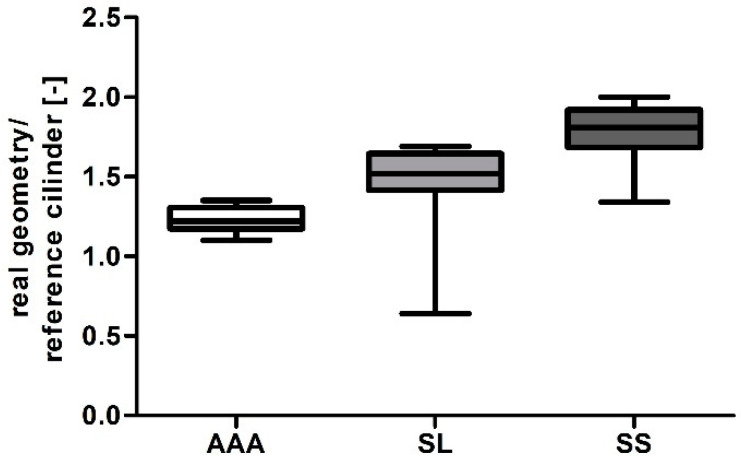
Bar chart representation of relation between a real geometry and a reference cylinder for abdominal aortic aneurysm (AAA), long stent-graft (SL), and short stent-graft (SS).

**Figure 4:**
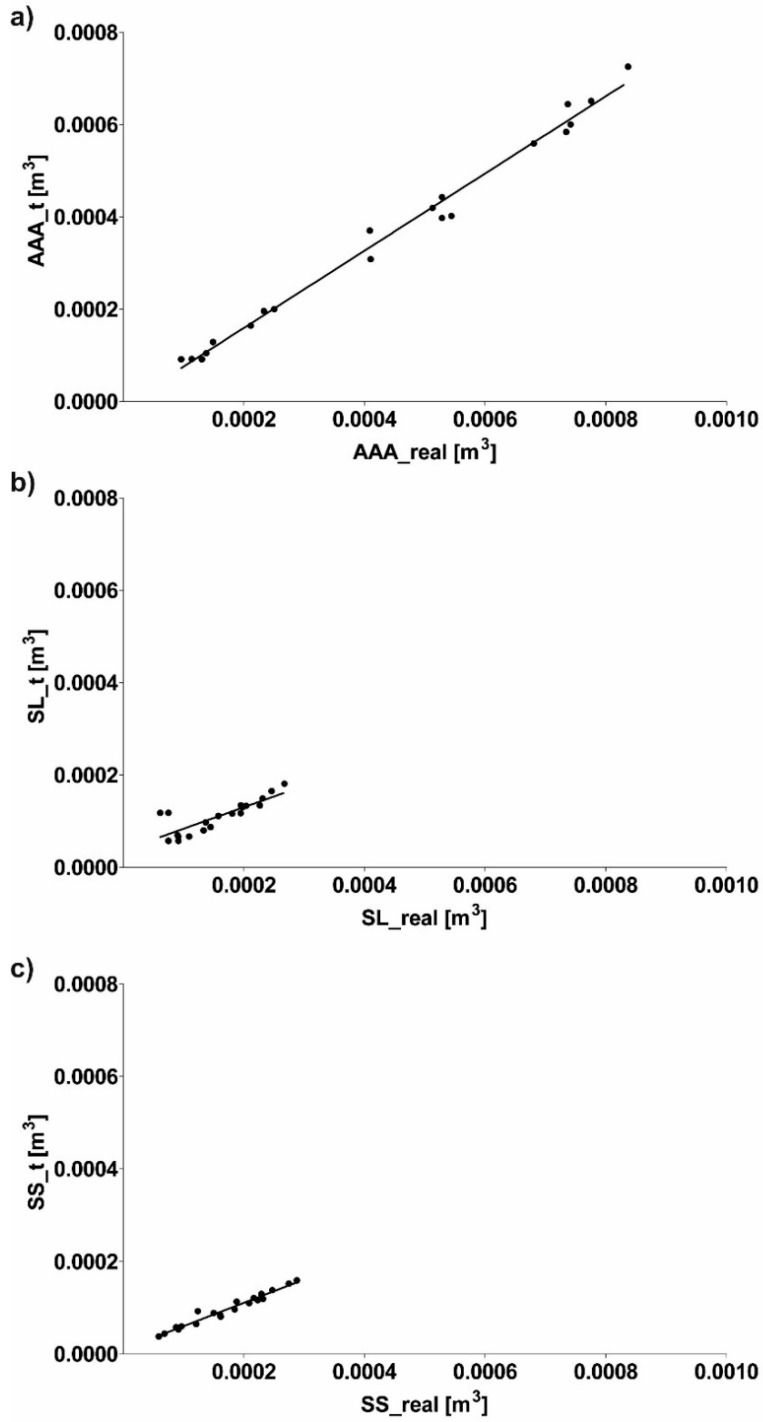
Scatterplot graphic representation of Pearson’s correlation factors rho [-] for the analyzed real geometry of (**a**) AAA in function of reference cylinder (rho = 0.9947), (**b**) SL in function of reference cylinder (rho = 0.8252), (**c**) SS in function of reference cylinder (rho = 0.9801). For all analyzes *p* < 0.001.

This section presents a numerical reconstruction of blood flow through 3D models of AAA and endovascular prosthesis. In the first step, relation between real geometry of AAA, long stent-graft (SL), short stent-graft (SS) and a reference cylinder was performed. Depending on the analyzed case shape factor was equal to 1.23 ± 0.09, 1.49 ± 0.14 and 1.76 ± 0.17 for AAA, SL and SS, respectively (Figure 3). Furthermore, Pearson’s rank correlation coefficients between real geometry and reference cylinders (Figure 4a–c) showed a positive correlation for AAA (rho = 0.9947, *p* < 0.001), SL (rho = 0.8252, *p* < 0.001) and SS (rho = 0.9801, *p* < 0.001). Each time real geometry had lower volume compare to the reference cylinder.

**Figure 5:**
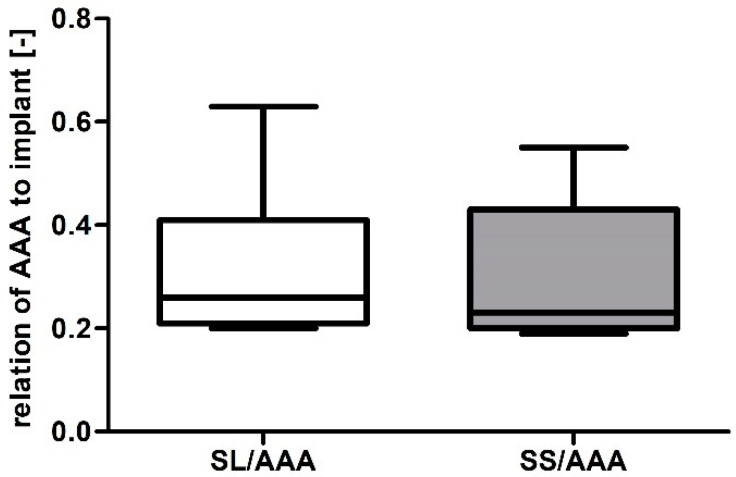
Bar chart representation of relation between aneurysm and stent-graft for abdominal aortic aneurysm (AAA), long stent-graft (SL), and short stent-graft (SS).

**Figure 6:**
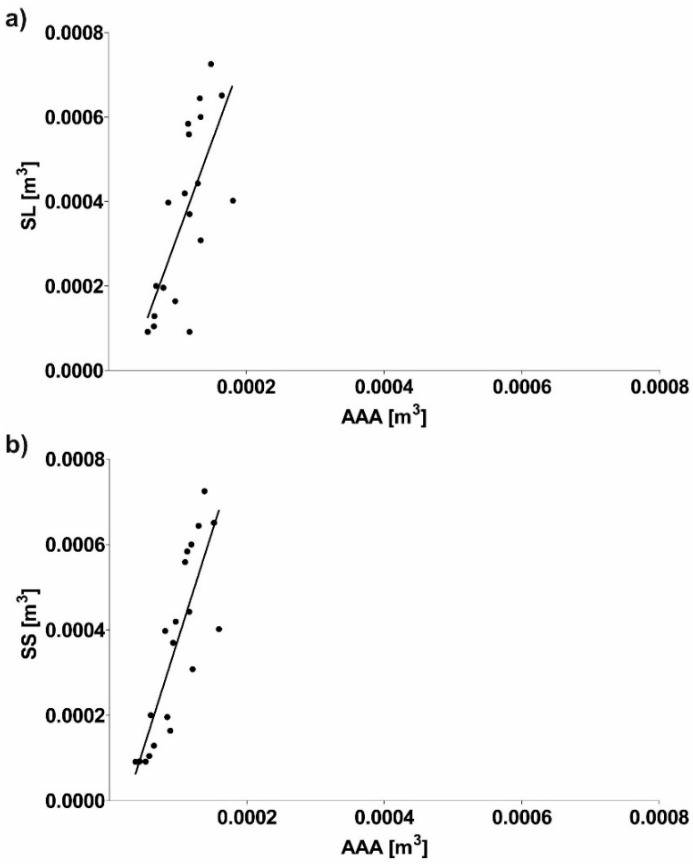
Scatterplot graphic representation of Pearson’s correlation factors rho [-] for the analyzed (**a**) SL in function of AAA (rho = 0.7402), (**b**) SS in function of AAA (rho = 0.8401). For all analyzes *p* < 0.002.

Next, the relation between AAA and implanted prosthesis was performed. Depending on the analyzed case, relation of AAA to an implant was equal to 0.33 ± 0.15 and 0.30 ± 0.13 for SL/AAA and SS/AAA, respectively (Figure 5). Furthermore, Pearson’s rank correlation coefficients were determined between implant and AAA (Figure 6a,b). There was a positive correlation between implant and AAA for SL/AAA (rho = 0.7402, *p* < 0.002) and SS/AAA (rho = 0.8401, *p* < 0.002). Increase of AAA volume indicated implementation of prosthesis with higher volume.

**Figure 7:**
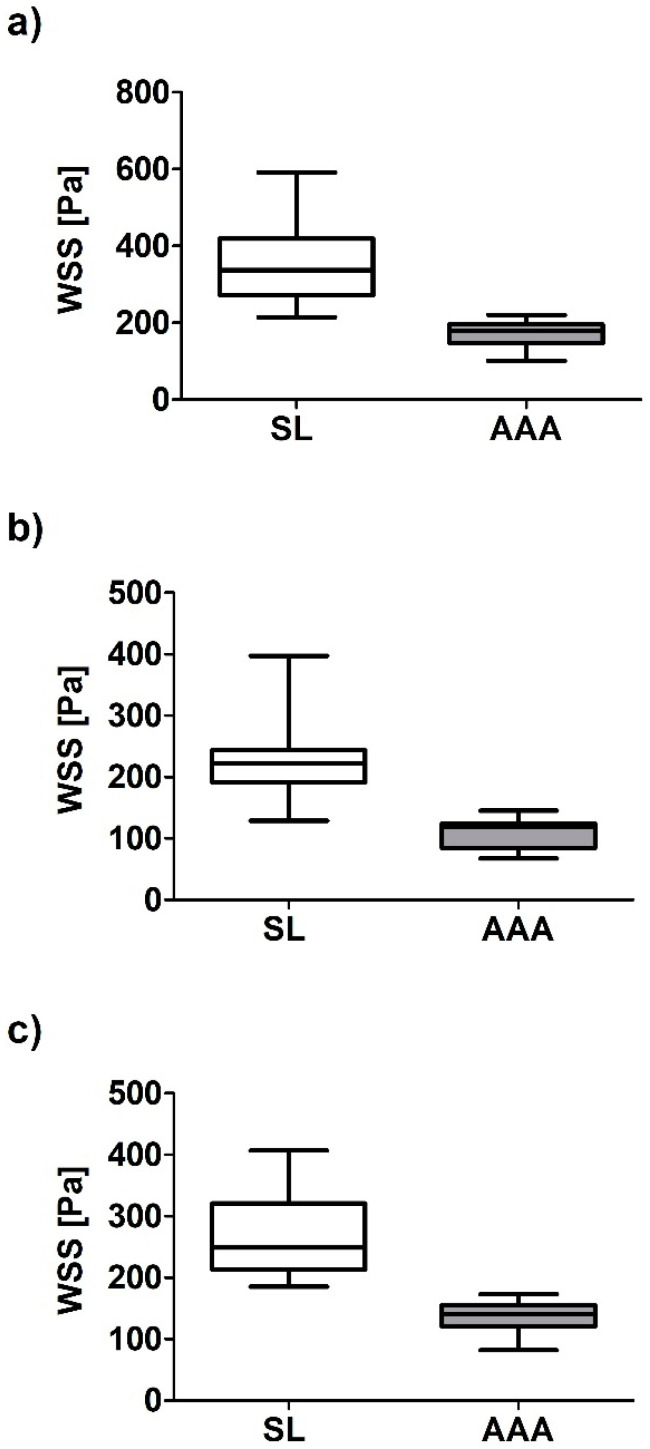
Bar chart representation of WSS value between aneurysm and stent-graft for abdominal aortic aneurysm (AAA) and long stent-graft (SL) for: (**a**) Sharp velocity profile, (**b**) flat velocity profile, (**c**) real velocity profile.

**Figure 8:**
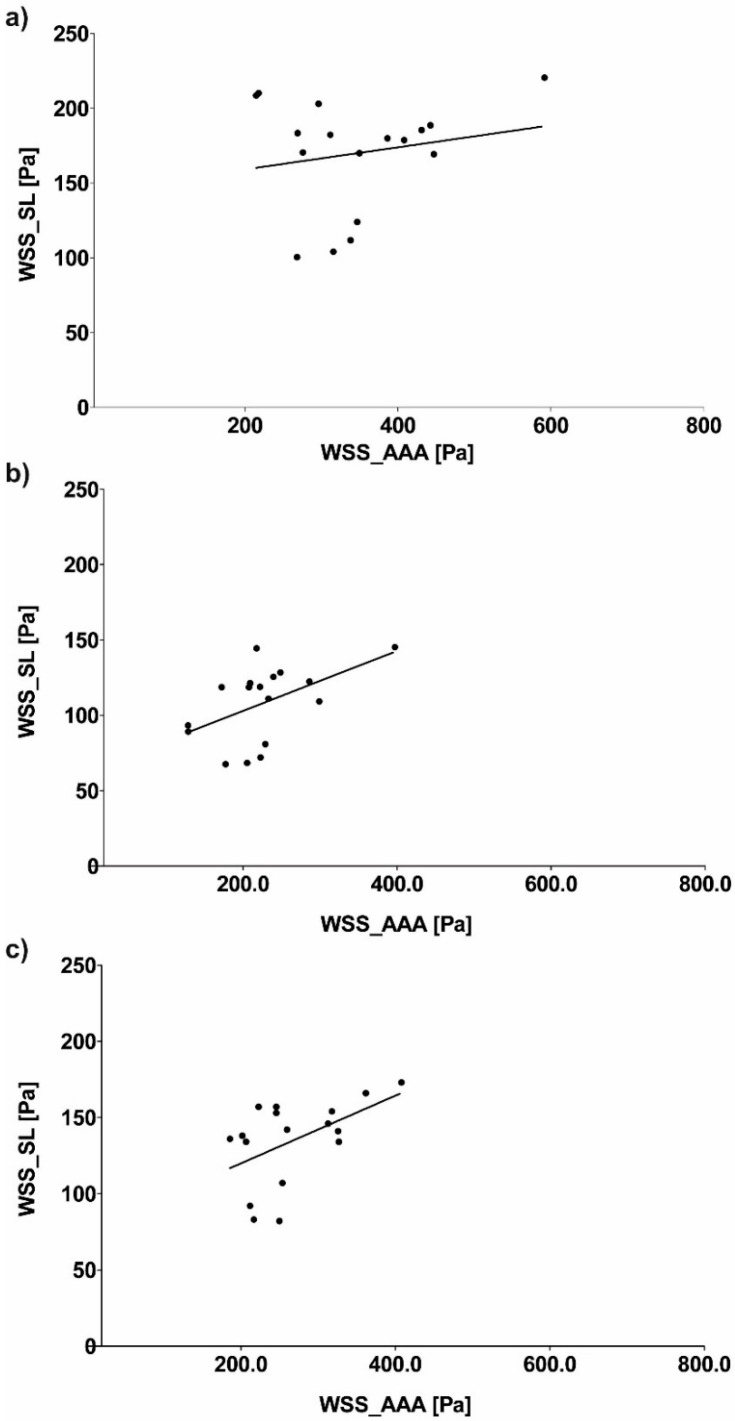
Scatterplot graphic representation of Pearson’s correlation factors rho [-] for the analyzed WSS_SL in function of WSS_AAA for: (**a**) Sharp profile (rho = 0.1889, *p* = 0.467), (**b**) flat profile (rho = 0.5052, *p* = 0.038), (**c**) real profile (rho = 0.5050, *p* = 0.038).

Next, relation of WSS values between analyzed geometries were calculated. WSS value between SL and AAA for all analyzed cases for sharp profile was equal to 367.85 ± 119.46 Pa and 199.74 ± 82.73 Pa for SL and AAA, respectively (Figure 7a). Moreover, WSS value between SL and AAA for all analyzed cases for flat profile was equal to 240.52 ± 81.43 Pa and 129.02 ± 58.14 Pa for SL and AAA, respectively (Figure 7b). Furthermore, WSS value between SL and AAA for all analyzed cases for real profile was equal to 282.10 ± 85.57 Pa and 163.40 ± 79.50 Pa for SL and AAA, respectively (Figure 7c). While Pearson’s rank correlation coefficients were determined between implant and AAA (Figure 8a–c). There was a weak positive correlation between SL/AAA for flat profile (rho = 0.5052, *p* = 0.038) and for SL/AAA for real profile (rho = 0.5050, *p* = 0.038) but not for SL/AAA for sharp profile (rho = 0.1889, *p* = 0.467).

**Figure 9:**
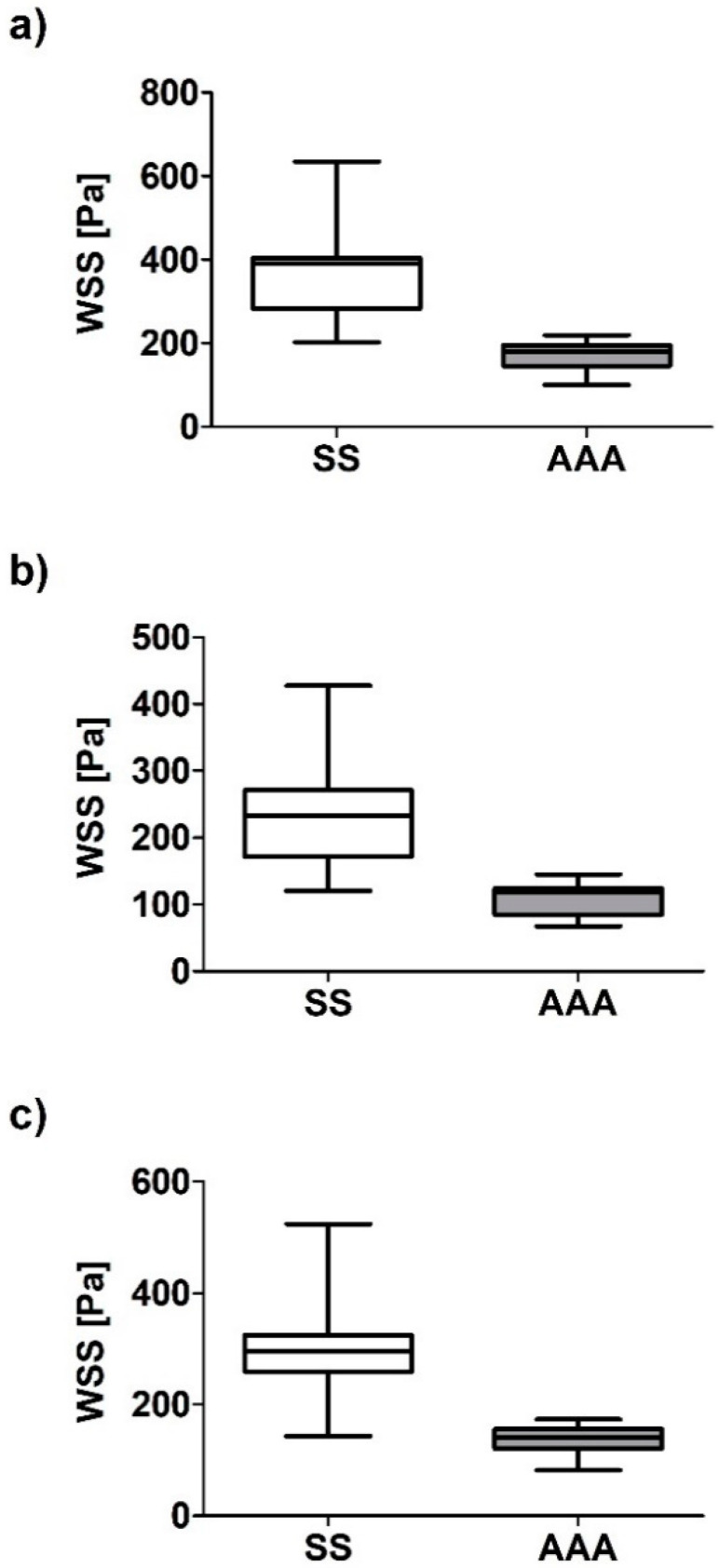
Bar chart representation of WSS value between aneurysm and stent-graft for abdominal aortic aneurysm (AAA) and short stent-graft (SS) for: (**a**) Sharp velocity profile, (**b**) flat velocity profile, (**c**) real velocity profile.

**Figure 10:**
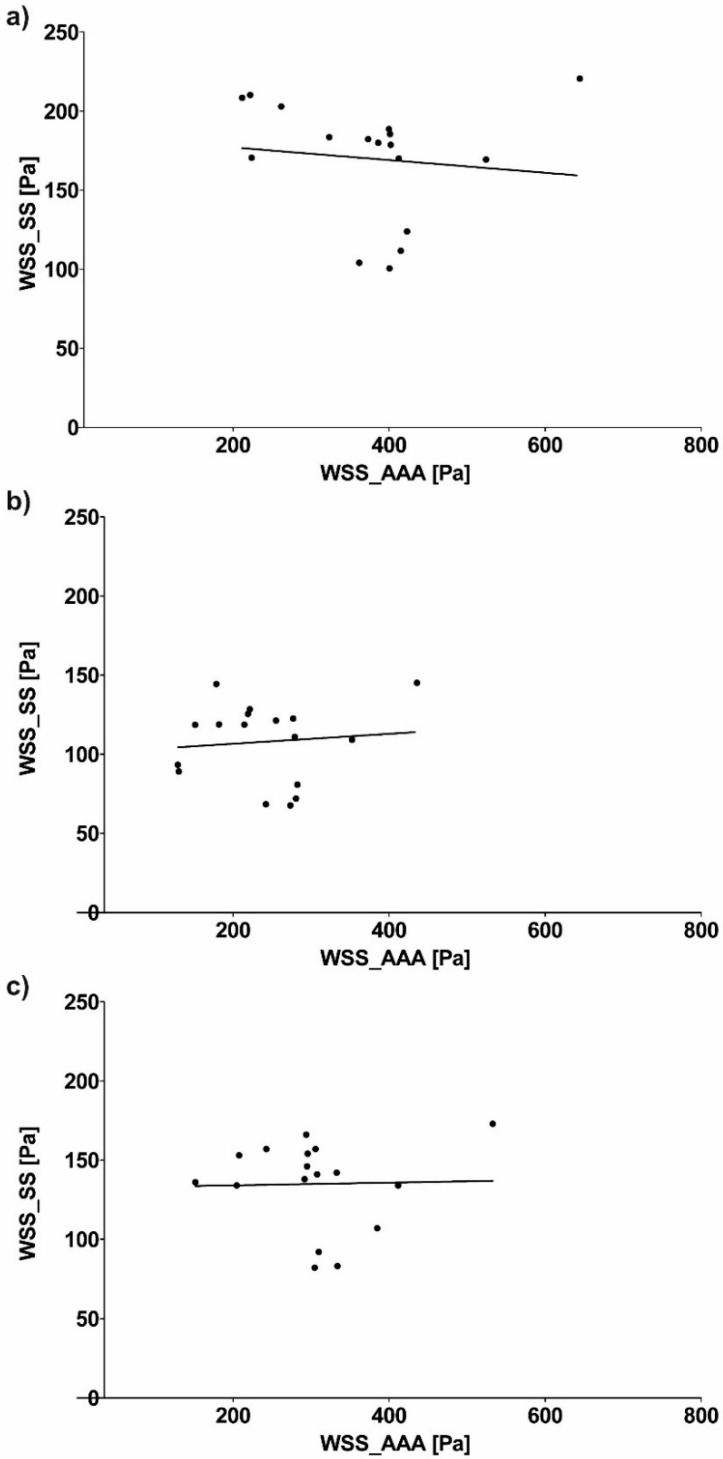
Scatterplot graphic representation of Pearson’s correlation factors rho [-] for the analyzed WSS_SS in function of WSS_AAA for: (**a**) Sharp profile (rho = −0.1171, *p* = 0.6544), (**b**) flat profile (rho = 0.098, *p* = 0.706), (**c**) real profile (rho = 0.0262, *p* = 0.920).

WSS value between SS and AAA for all analyzed cases for sharp profile was equal to 399.94 ± 147.23 Pa and 199.74 ± 82.73 Pa for SS and AAA, respectively (Figure 9a). Moreover, WSS value between SS and AAA for all analyzed cases for flat profile was equal to 257.55 ± 106.59 Pa and 129.02 ± 58.14 Pa for SL and AAA, respectively (Figure 9b). Furthermore, WSS value between SS and AAA for all analyzed cases for real profile was equal to 325.90 ± 124.60 Pa and 163.40 ± 79.50 Pa for SS and AAA, respectively (Figure 9c). While Pearson’s rank correlation coefficients were determined between implant and AAA (Figure 10a–c). No correlation between implant and AAA for SS/AAA for sharp profile (rho = −0.1171, *p* = 0.6544), for SS/AAA for flat profile (rho = 0.098, *p* = 0.706) or for SS/AAA for real profile (rho = 0.0262, *p* = 0.920).

**Figure 11:**
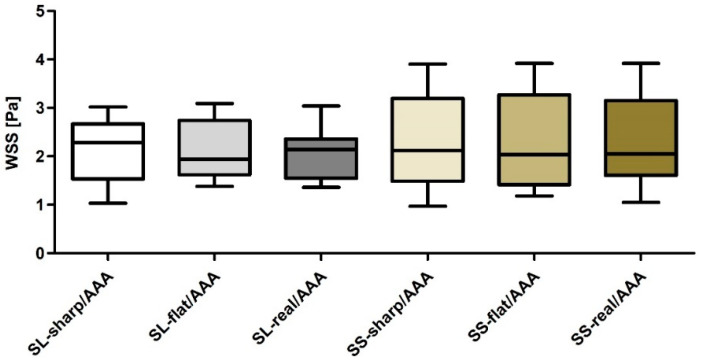
Bar chart representation of WSS value between aneurysm and stent-graft for abdominal aortic aneurysm (AAA), long stent-graft (SL), and short stent-graft (SS) for sharp velocity profile, flat velocity profile, real velocity profile.

**Figure 12:**
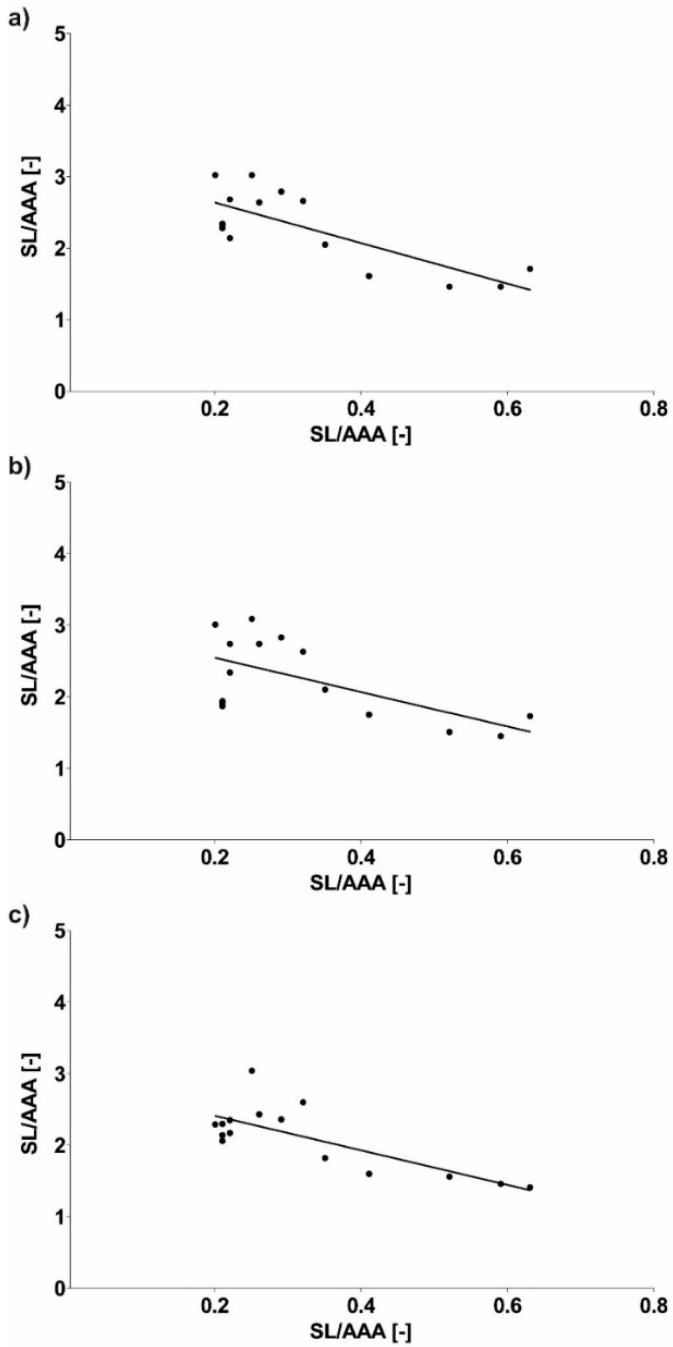
Scatterplot graphic representation of Pearson’s correlation factors rho [-] for the analyzed geometry of SL/AAA in function of WSS values for SL/AAA (**a**) for sharp profile (rho = −0.7747, *p* < 0.001), (**b**) for flat profile (rho = −0.6298, *p* = 0.011), (**c**) for real profile (rho = −0.7657, *p* < 0.001).

**Figure 13:**
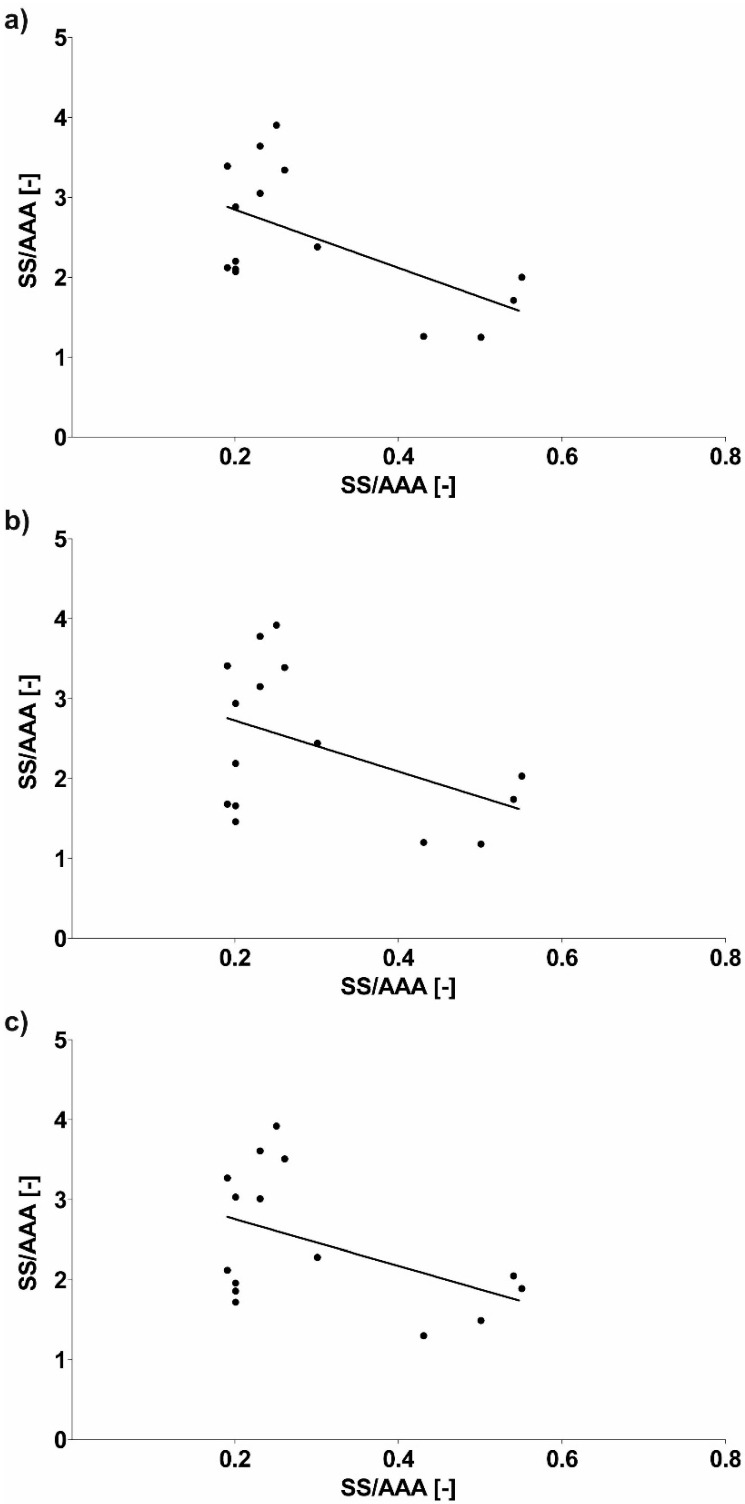
Scatterplot graphic representation of Pearson’s correlation factors rho [-] for the analyzed geometry of SS/AAA in function of WSS values for SS/AAA for (**a**) sharp profile (rho = −0.5887, *p* = 0.021), (**b**) flat profile (rho = −0.4537, *p* = 0.089), (**c**) real profile (rho = −0.4677, *p* = 0.078).

Next, geometrical description of AAA and implant in relation to WSS values was combined. It was observed that SL/AAA was equal to 2.28 ± 0.53, 2.24 ± 0.56 and 2.11 ± 0.46 for sharp profile, flat profile and real profile, respectively (Figure 11). While, for SS/AAA it was equal to 2.49 ± 0.83, 2.41 ± 0.95 and 2.47 ± 0.84 for sharp profile, flat profile and real profile, respectively (Figure 11). Furthermore, Pearson’s rank correlation coefficients were determined between implant and AAA. There was a strong negative correlation between SL/AAA and WSS values for SL/AAA for sharp profile (rho = −0.7747, *p* < 0.001) (Figure 12a), for flat profile (rho = −0.6298, *p* = 0.011) (Figure 12b) and for real profile (rho = −0.7657, *p* < 0.001) (Figure 12c). Additionally, there was a negative correlation between SS/AAA and WSS values for SS/AAA for sharp profile (rho = −0.5887, *p* = 0.021) (Figure 13a) but not for flat profile (rho = −0.4537, *p* = 0.089) (Figure 13b) and for real profile (rho = −0.4677, *p* = 0.078) (Figure 13c).

Finally, shape factor including spatial configuration and WSS value of implants localized in AAA was determined. For both types of implants linear function with negative slope was obtained (Table 1). Slope value for all analyzed stent-grafts was in a range from −2.405 ± 0.8226 to −2.835 ± 0.6418 for long stent-graft and from −2.918 ± 1.529 to −3.643 ± 1.388 for short stent-graft. While intersection with Y axis, representing a shape factor, was in range from 2.894 ± 0.1999 to 3.203 ± 0.2279 and from 3.338 ± 0.4974 to 3.572 ± 0.4514 for long stent-graft and short stent-graft, respectively. The shape factor was characterized by higher value for short stent-grafts compare to long stent-grafts. Moreover, it was observed that short stent-graft, after placement in the AAA, generated higher pushing forces compare to the long stent-graft. Therefore, it was observed that lower value of shape factor was presented for lower WSS values.

## 4. Discussion

Our study presents a novel approach to standardize the results of computer simulations basing on spatial configuration of different type of stent-grafts in relation to AAA. Computational model of blood hemodynamics together with US-Doppler and AngioCT data allowed investigation of blood distribution. The operation of the algorithm was positively evaluated in house (data not yet published) by radiologists from the Department of Radiology and Diagnostic Imaging in Barlicki Hospital in Lodz, Poland. They applied the proposed mathematical tool to assess the impact of endovascular prosthesis placed in AAA on blood hemodynamic.

Using the proposed algorithm, it is possible to estimate how the spatial configuration of the stent-graft may disturb blood hemodynamics, leading to an increase in WSS value, which may result in an aneurysm rupture. There is a significant impact of endovascular prosthesis construction as presented by [39]. Pintoux et al. observed also that endovascular prostheses implanted in AAA with short neck, wide sack and widened iliac arteries are more prone to migration contrary to the endovascular prostheses that are anchored in long neck and normal iliac arteries [40]. While, Avgerinos et al. noticed that endovascular prostheses composed of several elements are more prone to migration [41]. It was in line with our study, where we noticed that spatial configuration of endovascular prostheses affected the value of pushing forces. A comparison of different endovascular prostheses geometry enabled us to estimate the effect of changes in blood hemodynamic on the value of pushing forces formed in the region of proximal fixation. Raben et al. observed that wider bifurcation angles increases area of low flow and recirculation [42], while Yu and Kwon observed that endovascular prosthesis’s design parameters such as number of strands, or strut angle have also significant influence on implant’s migration risk. They also observed that an ideal endovascular prosthesis should have higher strut angle [43].

Moreover, WSS is not the only factor that induce endovascular prosthesis migration [44]. Furthermore, Ribeiro et al. observed that the stent-graft’s strut thickness is one of the most significant design parameters, and an increase in thickness indicates deceleration of blood flow and recirculation zones [45]. Nowadays, many studies connected with computer simulations focus on assessing the blood hemodynamic in the vessels after endovascular prosthesis placement in AAA. An integrated mathematical, physics, and clinical approach have improved understandings and applications of interventional cardiology from stent design to implantation [46]. Lamooki et al. noticed that computer modeling of stent expansion is critical for preventing unrealistic bulging effects and thus should be considered in virtual flow diverter deployment algorithms [47]. Patient specific AAA blood hemodynamic simulation is a promising tool that may provide important information for understanding hemodynamic factors involved in AAA repair and potentially provide data for clinical decision making [48]. In our study, as well as in Harrison et al., no comparison between the effects of the different mechanical properties of the patch and of the native artery in terms of disturbed flow [49]. We have assumed rigid walls in our computations, so that the only factor which in fact drives the results is the geometry.

### Limitations to the Study

Although our study demonstrates the novel methodology for the description of WSS estimation after stent-graft placement in the AAA it has some limitations. Small sample size could influence the obtained results. However, the patients were carefully selected to uniform the group, hence we believe the obtained results may be applicable to similar cases. Secondly, simulations accuracy depends on the resolution of CTA data. The higher the resolution the better is the three-dimensional reconstruction and the results of WSS distribution. In the next step we would like to extend this study and analyze wider group of patients. Moreover, it is essential to determine what extent changes in blood hemodynamic in the area below the implant, can affect stability of the stent-graft fixation. Therefore, preparation of precise recommendations for correct location of the stent-graft or blood hemodynamic below the implant would be an advantage of computer simulations. Furthermore, in order to verify a reliability of the CFD model, the number of analyzed patients should be increased.

## 5. Conclusions

In summary, the proposed self-made algorithm demonstrated its efficiency and usefulness in estimating the WSS values after endovascular prosthesis placement inside abdominal aortic aneurysm. Comparison of the stent-grafts’ long and short type with AAA geometries and its reference cylinders, generated on their basis, allowed the analysis of the implants’ spatial configuration as well as the risk of movement by acting of pushing forces.

Our study indicated that short stent-graft, after placement in the AAA, generated higher drag forces compare to the long stent-graft. Furthermore, it was observed that lower value of shape factor was presented for lower WSS values. Therefore, it was observed that the shape factor was characterized by higher value for short stent-grafts compare to long stent-grafts.

We believe that our algorithm prepared in this study may become a useful non-invasive quantitative tool for radiologists and surgeons for the characterization of blood hemodynamic in the area of AAA before and after stent-graft placement as well as value of drag force acting on the endovascular prosthesis after placement inside AAA. However, further studies are necessary to confirm its usefulness in clinical practice.

## Figures and Tables

**Table 1:** Shape factor describing long and short stent-graft with the use of linear regression.

Type of Stent-Graft	Slope	Y-Intercept
SL—long stent-graft	−2.835 ± 0.6418	3.203 ± 0.2279
	−2.405 ± 0.8226	3.027 ± 0.2921
	−2.416 ± 0.5628	2.894 ± 0.1999
SS—short stent-graft	−3.643 ± 1.388	3.572 ± 0.4514
	−3.184 ± 1.735	3.360 ± 0.5643
	−2.918 ± 1.529	3.338 ± 0.4974
